# Data on the abundance of the banana weevil *Cosmopolites sordidus* and of the earwig *Euborellia caraibea* in bare soil and cover crop plots

**DOI:** 10.1016/j.dib.2016.04.056

**Published:** 2016-04-27

**Authors:** Dominique Carval, Rémi Resmond, Raphaël Achard, Philippe Tixier

**Affiliations:** a Campus Agroenvironnemental Caraibes, CIRAD, UPR GECO, Petit Morne, F-97285 Le Lamentin, Martinique, France; b CIRAD, UPR GECO, F-34398 Montpellier, France; c Departamento de Agricultura y Agroforesteria, CATIE, CR-30501 Turrialba, Costa Rica

## Abstract

The data presented in this article are related to the research article entitled “Cover cropping reduces the abundance of the banana weevil *Cosmopolites sordidus* but does not reduce its damage to the banana plants” (Carval et al., in press) [1]. This article describes how the abundance of the banana weevil, *Cosmopolites sordidus*, and the abundance of the earwig *Euborellia caraibea* were affected by the addition of a cover crop. The field data set is made publicly available to enable critical or extended analyzes.

**Specifications Table**

**Table:** 

Subject area	*Biology*
More specific subject area	*Field survey of a banana pest*
Type of data	*Table, figure, text file*
How data was acquired	*Field survey*
Data format	*Raw, filtered*
Experimental factors	*Features of the banana weevils (maturity) and spatial coordinates of samples were recorded*
Experimental features	*Field data: trap catches of weevils and earwigs in* 3 *bare soil and* 3 *cover crop plots of banana plants*
Data source location	14°39′45.04″N; 60°59′59.08″W
Data accessibility	*Data is available with this article*

**Value of the data**

• The data is valuable for other researchers working on this pest or in similar scientific field.
• This data offers the opportunity to other researchers to compare with their own datasets.
• This data enables other researchers to independently verify or extend statistical analyses.

## 1 Data

**Fig. 1:**
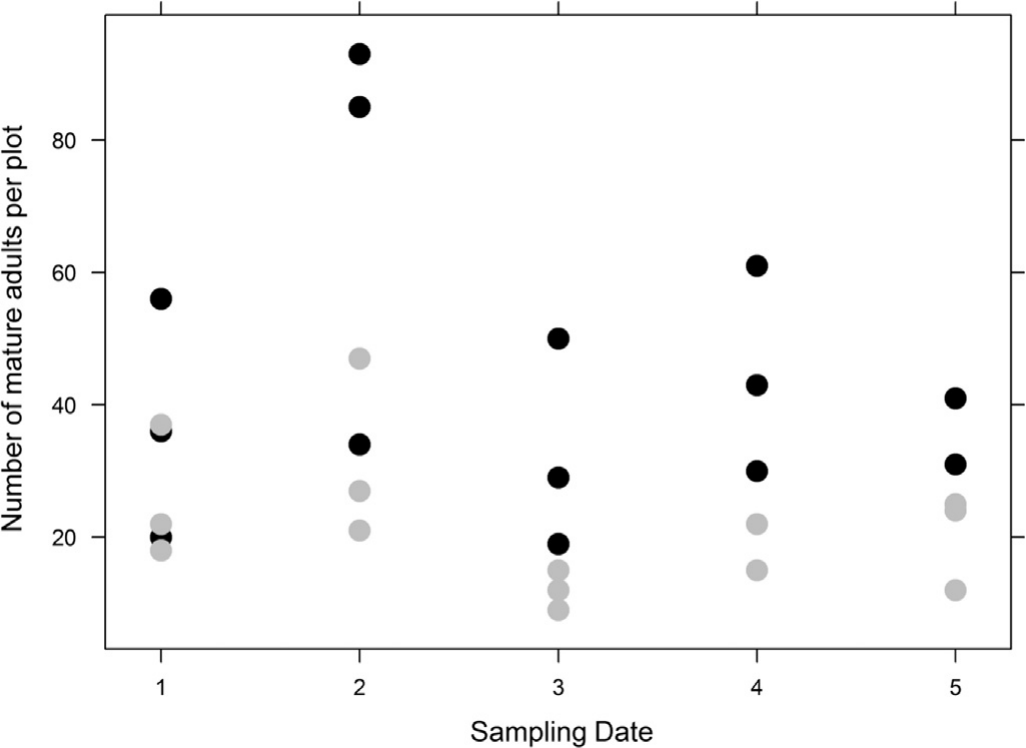
Number of the mature adults of *C. sordidus* per plot as affected by sampling date and soil cover. Black points: bare soil plots; gray points: cover crop plots.

**Fig. 2:**
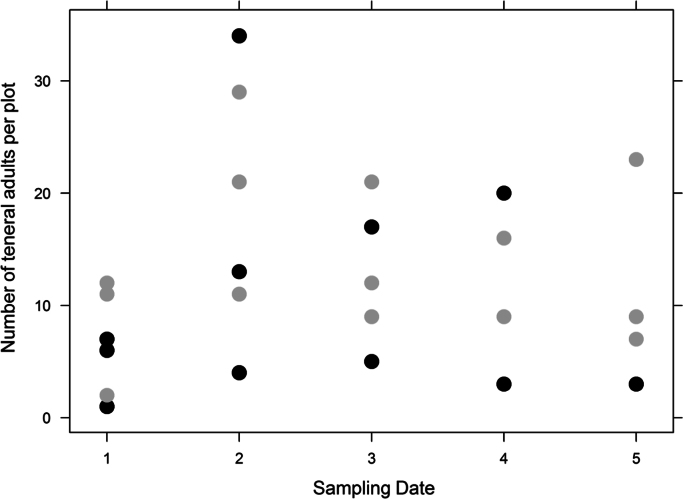
Number of the teneral adults of *C. sordidus* per plot as affected by sampling date and soil cover. Black points: bare soil plots; gray points: cover crop plots.

**Fig. 3:**
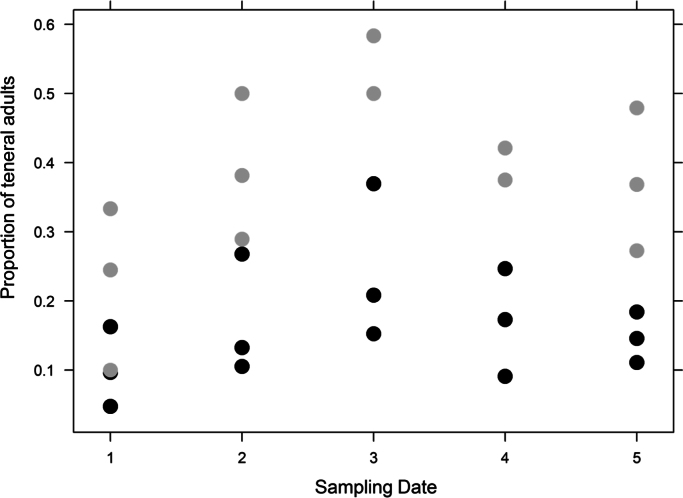
Proportion of teneral adults relative to all *C. sordidus* adults per plot as affected by sampling date and soil cover. Black points: bare soil plots; gray points: cover crop plots.

**Fig. 4:**
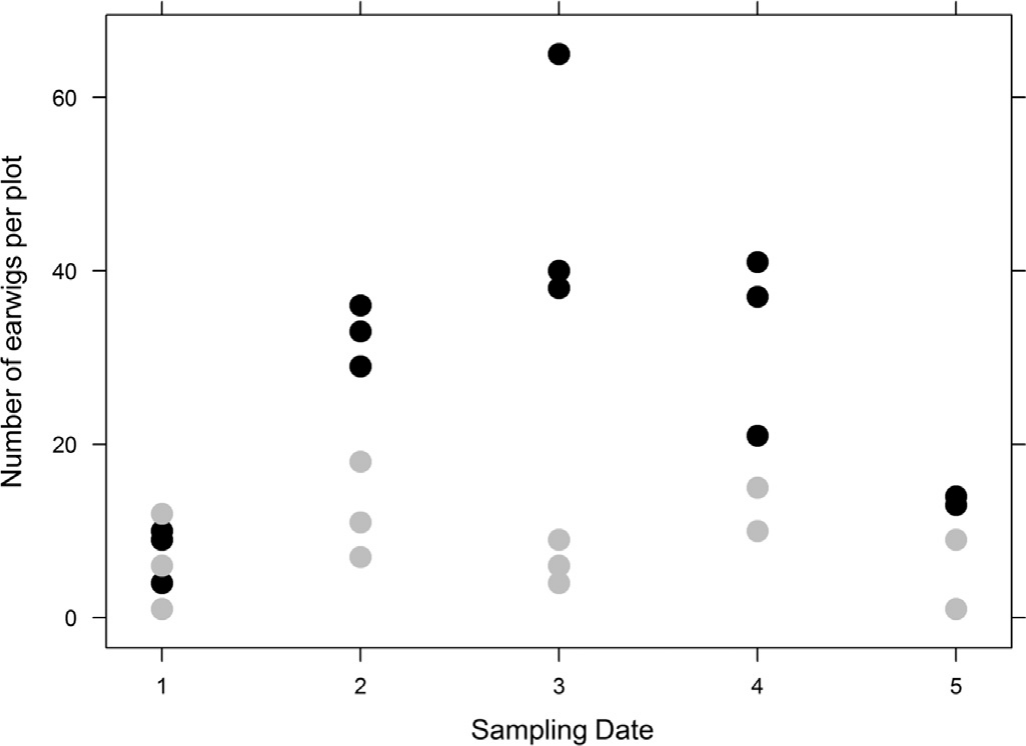
Number of the earwigs per plot as affected by sampling date and soil cover. Black points: bare soil plots; gray points: cover crop plots.

The dataset provided in this article gives information on the abundance of the banana pest *Cosmopolites sordidus* and the predator *Euborellia caraibea* in banana plots with or without the cover crop *Paspalum notatum*. Fig. 1, Fig. 2, Fig. 3, Fig. 4 display details on the population dynamics and composition (teneral vs. mature weevils) across sampling dates. Table 1 provides the corresponding filtered data. Appendix A contains raw data with spatial coordinates.

## 2 Experimental design, materials and methods

The experiment was conducted in six plots were established, each with an area of 361 m² and with 49 banana plants (Cavendish Grande Naine cultivar). The cover crop *P*. *notatum* was planted in three of the plots (CC soil type plots) while the other three plots were maintained with bare soil (BS soil type plots) through the use of herbicide (glyphosate). Banana weevil and earwig abundances were estimated with pseudostem traps deposited at the basis of each banana plant. We discriminated between teneral adult weevils and mature weevils based on exoskeleton color [2]. The data was recorded at the plant scale and associated with spatial coordinates of each banana plant.

The data presented in this article are related to the research article entitled "Cover cropping reduces the abundance of the banana weevil *Cosmopolites sordidus* but does not reduce its damage to the banana plants" (Carval et al., in press) [1].

## Figures and Tables

**Table 1:** Abundances of the earwig *E*. *caraibea* and of the weevil *C*. *sordidus* (total, teneral and mature adult numbers) per plot at each sampling date. BS: bare soil plots; CC: cover crop plots.

**Sampling date**	**Plot**	**Soil type**	**Number of earwigs**	**Number of weevils**	**Number of teneral weevils**	**Number of mature weevils**	**Proportion of teneral weevils (%)**
1	P1	BS	9	43	7	36	16.28
2	P1	BS	29	98	13	85	13.27
3	P1	BS	38	59	9	50	15.25
4	P1	BS	41	81	20	61	24.69
5	P1	BS	13	48	7	41	14.58
1	P2	CC	12	33	11	22	33.33
2	P2	CC	11	76	29	47	38.16
3	P2	CC	9	36	21	15	58.33
4	P2	CC	10	24	9	15	37.50
5	P2	CC	1	19	7	12	36.84
1	P3	BS	10	62	6	56	9.68
2	P3	BS	36	127	34	93	26.77
3	P3	BS	40	46	17	29	36.96
4	P3	BS	37	52	9	43	17.31
5	P3	BS	14	27	3	24	11.11
1	P4	CC	6	49	12	37	24.49
2	P4	CC	18	38	11	27	28.95
3	P4	CC	4	18	9	9	50.00
4	P4	CC	15	38	16	22	42.11
5	P4	CC	1	33	9	24	27.27
1	P5	BS	4	21	1	20	4.76
2	P5	BS	33	38	4	34	40.53
3	P5	BS	65	24	5	19	20.83
4	P5	BS	21	33	3	30	9.09
5	P5	BS	13	38	7	31	18.42
1	P6	CC	1	20	2	18	10.00
2	P6	CC	7	42	21	21	50.00
3	P6	CC	6	24	12	12	50.00
4	P6	CC	15	24	9	15	37.50
5	P6	CC	9	48	23	25	47.92

## Footnotes

Appendix A Supplementary data associated with this article can be found in the online version at doi:10.1016/j.dib.2016.04.056.
