# Supplementary material for: Data on the abundance of the banana weevil Cosmopolites sordidus and of the earwig Euborellia caraibea in bare soil and cover crop plots
Source: Data Brief. 2016 Apr 27;7:1565–9. doi: 10.1016/j.dib.2016.04.056 (PMC4865667; doi:10.1016/j.dib.2016.04.056)
Supplement: Supplementary file 1 — Supplementary material [file mmc1.docx]

DIB-D-16-00276

Title: **Data on the abundance of the banana weevil *Cosmopolites sordidus* and of the earwig *Euborellia caraibea* in bare soil and cover crop plots**

Authors: Dominique Carval; Resmond Rémi; Raphaël Achard; Philippe Tixier

Conflict of interest: none

On the behalf of authors,

Dominique Carval
